# Supplementary material for: Internal protein motions in molecular-dynamics simulations of Bragg and diffuse X-ray scattering
Source: IUCrJ. 2018 Jan 25;5(Pt 2):172–81. doi: 10.1107/S2052252518000519 (PMC5947722; doi:10.1107/S2052252518000519)
Supplement: Supplementary file 1 [file m-05-00172-sup1.pdf]

IUCrJ

Volume 5 (2018)

Supporting information for article:

Internal protein motions in molecular-dynamics simulations of Bragg and diffuse X-ray scattering

Michael E. Wall

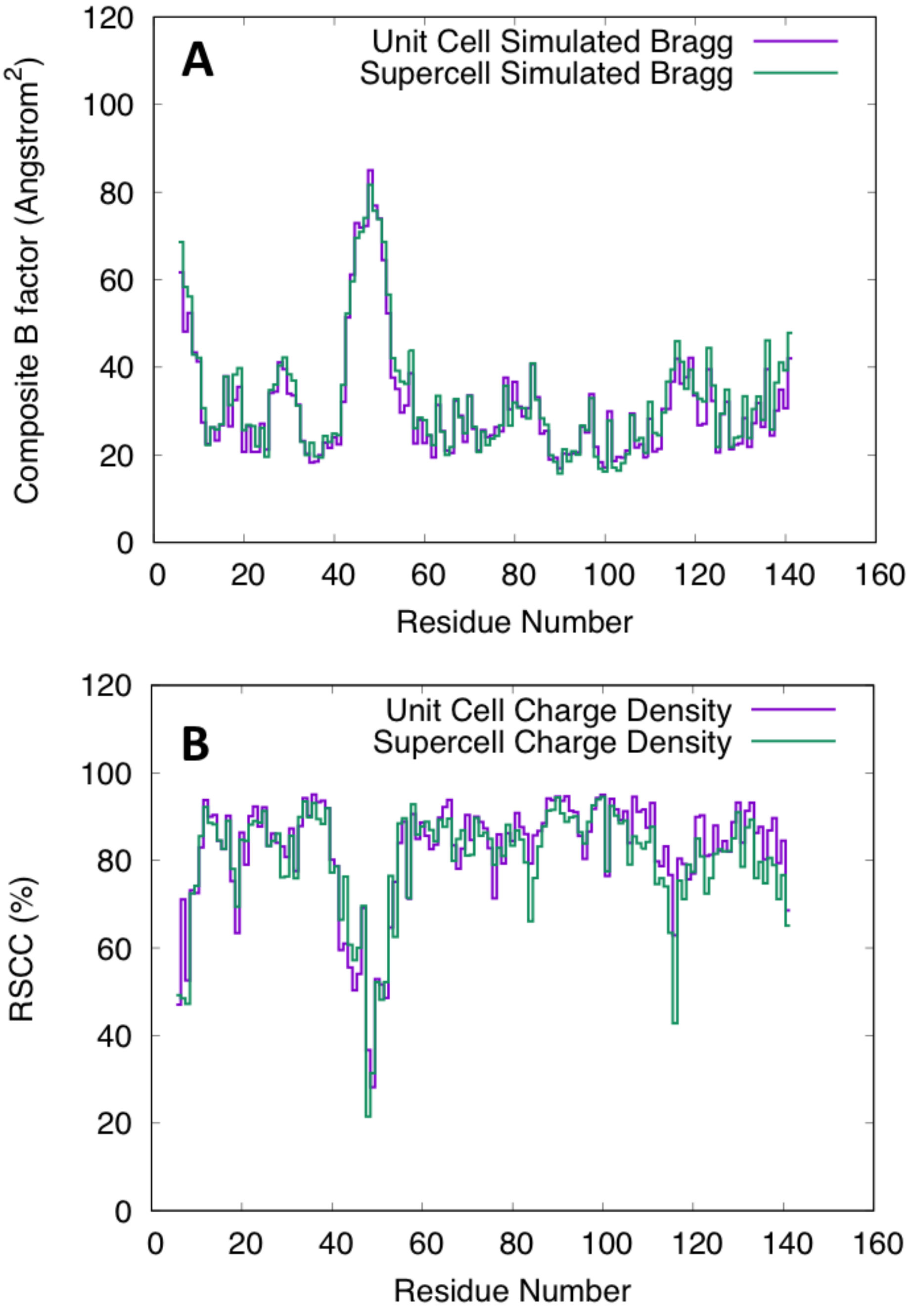

Figure S1 Comparison of Bragg analysis results for the unit cell vs. supercell simulations. (A) Residue-wise comparison between the B factors from the crystal structure (purple) and simulated average structures from the unit cell (green) and supercell (cyan) simulations. (B) Residue-wise RSCC computed between the crystal structure and the charge density from the supercell (green) and unit cell (cyan) simulations.
